# A mutation in the *UBIAD1* gene in a Han Chinese family with Schnyder corneal dystrophy

**Published:** 2011-10-15

**Authors:** Chunyu Du, Ying Li, Lili Dai, Lingmin Gong, Chengcheng Han

**Affiliations:** Department of Ophthalmology, Harbin Medical University the 2nd Affiliated Hospital, Harbin, China

## Abstract

**Purpose:**

To identify the molecular defect in the UbiA prenyltransferase domain containing 1 (*UBIAD1*) gene in a four-generation Chinese family with Schnyder corneal dystrophy (SCD).

**Methods:**

A four-generation Chinese family with SCD and 50 unrelated normal individuals as controls were enrolled in. The complete ophthalmic examination was performed and blood samples were taken for subsequent genetic analysis. Mutation screening of *UBIAD1* was performed by polymerase chain reaction (PCR) based DNA sequencing.

**Results:**

The missense mutation N102S in *UBIAD1* was identified in this pedigree from the mainland of China for the first time. The molecular defect cosegregates with the affected individuals, whereas not found in unaffected family members or normal controls.

**Conclusions:**

The nonsynonymous mutation, N102S, in *UBIAD1* detected in this family confirms that it is a mutation hot spot not only in Caucasian but also in Chinese. This finding adds support to the proposal that N102S has been independently mutated and argues against the likelihood of a founder effect.

## Introduction

Schnyder corneal dystrophy (SCD; OMIM 121800) is a rare autosomal dominant disease characterized by bilateral and usually symmetric cholesterol and lipid deposits in the corneal stroma with or without crystals. SCD results in progressive corneal opacification, loss of visual acuity (especially photopic vision [1]), and eventually corneal sensation or glare. The clinical manifestation of this dystrophy, while variant, is most commonly in an axially distributed, annular, or discoid pattern. The appearance of the cornea can be predicted based on age. Although SCD has also been known as Schnyder crystalline corneal dystrophy, only 54% of patients have corneal crystals [1]; the nomenclature itself confounded the ability to make an accurate diagnosis. Recently, the International Committee for the Classification of Corneal Dystrophies (IC3D) [2] renamed the dystrophy Schnyder corneal dystrophy to clarify that crystalline deposition was not integral to the diagnosis. Other systemic findings associated with SCD are hypercholesterolemia and genu valgum, which are thought to be independent traits and are found in approximately 66% [3-5] and 4% [1] of affected patients, respectively.

In the past decade, significant advances have been made in determining the genetic basis of SCD. Shearman et al. [6] first localized SCD to chromosome 1p36 through the linkage analysis in two large Swedish-Finnish families. In 2007, Orr et al. [7] and Weiss et al. [8] independently verified that the mutational UbiA prenyltransferase domain containing 1 gene (*UBIAD1*) caused SCD. Thus, it is generally postulated that the onset of SCD is associated with mutations in *UBIAD1* caused by base substitution. To date, 22 different mutations (only in exons 1 and 2) have been reported: A97T [9], G98S [10], Y174C [11], N102S [7,8,12,13], D112G [7], D112N [9], D118G [13], R119G [7,12], L121V [12,13], L121F [14], V122E [9], V122G [9], S171P [13,15], T175I [7,13], G177R [8,13], K181R [11], G186R [13], L188H [9], N232S [7], N233H [11], D236E [13], and D240N [16]. Studies of the genetic basis of SCD demonstrated that all mutations in the *UBIAD1* gene were missense mutations, with N102S postulated to be a hot spot in Caucasians because it was the most frequent mutation [13]. SCD results from one of the numerous mutations in *UBIAD1* [7,8]. To our knowledge, the present study contains the first description of the mutation N102S in the Han Chinese in mainland China.

## Methods

### Patients and controls

**Figure 1:**
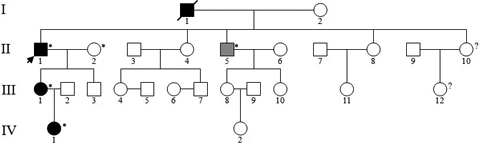
Pedigree of the proband’s family with Schnyder corneal dystrophy. Black symbols, gray symbols, and unfilled symbols represent individuals of affected members, indeterminate phenotype, and unaffected members, respectively. Question marks indicate individuals of unknown affected status, and arrow indicates the proband. Asterisks indicate individuals in whom DNA analysis were performed. Deceased family member was denoted by slash.

This study was approved by the Institutional Review Board of Harbin Medical University (Harbin, China), and informed consent was obtained from each participant before participation. All subjects underwent a complete eye examination, including uncorrected visual acuity (UCVA), best-corrected visual acuity (BCVA), pupillary reaction, intraocular pressure, motility, slit-lamp examination, corneal sensitivity testing, and fundus examination. Corneal sensation was tested by lightly touching the cornea with a wisp of cotton from a cotton swab. We studied a four-generation Chinese family from northeastern mainland China with SCD (Figure 1); the family’s ethnic background was not Caucasian. Three patients, ten unaffected family members, and fifty healthy unrelated normal controls were recruited in this research. In addition, each subject with SCD underwent laboratory examinations including routine blood tests, biochemical examination of the blood, physical examination, and radiography of the knee joints.

### Genetic Analysis

Venipuncture was performed for DNA collection, and peripheral blood (3 ml) was drawn from each subject. Genomic DNA was isolated from the peripheral blood leukocytes using the TIANamp Blood DNA Kit (Tiangen Biotech Co. Ltd, Beijing, China), following the manufacturer’s instructions. Exons 1 and 2 of *UBIAD1* were amplified by polymerase chain reaction (PCR) using a 50-ml reaction volume that contained 10× PCR buffer, 0.2 mM of each deoxyribonucleotide triphosphate, 2 μl of 1 mM of each primer, 0.5 units of Taq polymerase (Takara Biotechnology Co. Ltd, Dalian, China), and 10–200 ng of genomic DNA. Primers for the two coding exons of *UBIAD1* were *UBIAD1*: 1F-CTC GTG GGG TGT AAG ACC CAC TT, 1R-GCG GCT TAA ATT AGA AAG CCA CCT; 2F-AGT GCC CAC CTG CAC AGT CTA AG, 2R-CAA ACT GGG CAG CTC CTT TAC AA [12]. The iCycler Thermal Cycler (Bio-Rad, Hercules, CA) was used for the thermocycling procedure. The protocol for amplification reactions was as follows: denaturation at 95 °C for 5 min, then followed by 35 cycles at 94 °C for 30 s, 63 °C to 65 °C for 30 s, 72 °C for 40 s, and the terminal extension step at 72 °C for 8 min. The annealing temperatures are 63 °C for exon 1 and 65 °C for exon 2. 2% agarose gel was used to detect PCR products, and subsequently the PCR products were purified with a TIANgel Midi Purification Kit (Tiangen Biotech Co. Ltd). For direct sequencing via an ABI BigDye Terminator Cycle Sequencing kit v3.1 (ABI Applied Biosystems, Foster City, CA), the PCR products were sequenced by an ABI 3100 Genetic Analyzer (Applied Biosystems). Nucleotide sequences of PCR products were manually compared with gene annotation from GeneBank (NM_013319.2).

## Results

### Clinical findings

**Figure 2:**
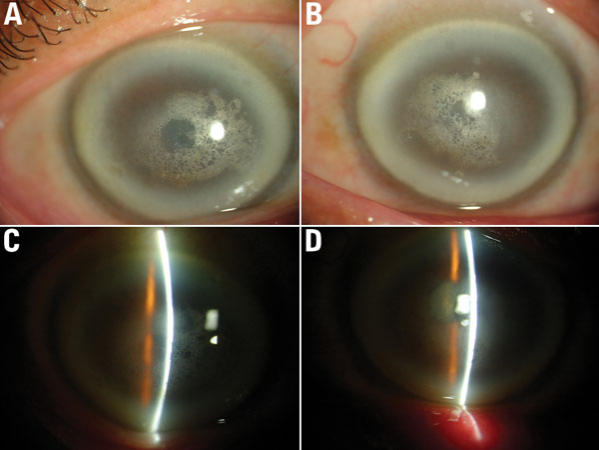
The proband’s corneal findings. Corneal photos of the proband demonstrate central and paracentral crystalline deposits, central and midperipheral haze, and arcus lipoides in a 57 year old male. **A** and **B**: External photographs of OD and OS. **C** and **D**: Slit-lamp photograph of OD and OS, demonstrate subepithelial crystalline deposits.

**Figure 3:**
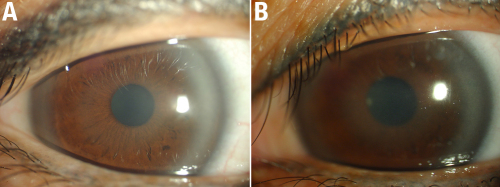
Corneal findings of the proband’s daughter. External photographs of the cornea of the proband’s daughter, a 34 year old female, III:1, with central discoid haze, midperipheral clouding, and peripheral arcus lipoides, without crystals. **A**: OD and **B**: OS.

**Figure 4:**
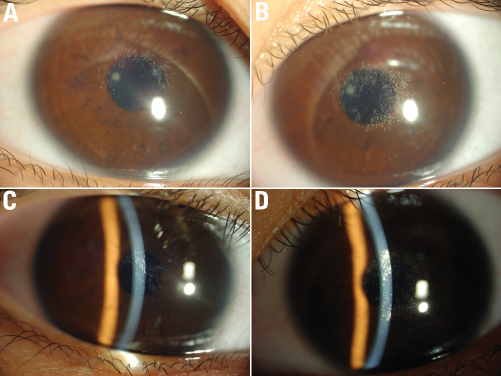
Corneal findings of the proband’s granddaughter. The corneas of an 11 year old female, IV:1, with almost complete circle of crystalline deposition that appears to be not symmetric. The crystals in left eye (**B**, **D**) is more than that in right eye (**A**, **C**).

The proband (Figure 1) was a 57-year-old male who was referred to our center due to his complaint that he had been “seeing things hazily” for two decades. UCVA was 20/40 OD, 20/200 OS, and BCVA was 20/30 OD, 20/80 OS. Slit-lamp examination revealed central and paracentral subepithelial crystalline deposits, central and midperipheral haze, and arcus lipoides (Figure 2). Corneal sensation was reduced in the right eye and normal in the left eye. Pupillary reaction, intraocular pressure, and motility were normal, yet the fundus of both eyes could not be clearly observed. Knee valgus was not found through knee examination. The blood biochemical examination showed elevated levels of serum total cholesterol and low levels of calcium. He had a history of ocular contusion injuries without treatment 25 years ago, and no history of coronary heart disease or cerebrovascular disease. The proband’s 34-year-old daughter (Patient III:1) had good vision (20/15 OD and 20/20 OS); nevertheless, slit-lamp examination demonstrated bilateral central discoid haze, midperipheral clouding, and peripheral arcus lipoides without crystals (Figure 3). Pupillary reaction, motility, corneal sensation, intraocular pressure, fundus examination, and knee examination were all normal. The only abnormal laboratory reading was a slight decrease in fasting plasma glucose recorded during biochemical examination of the blood. Patient IV: 1 (Figure 1) was an 11-year-old girl with good UCVA: 20/20 OD and 20/15 OS. Slit-lamp examination revealed an almost complete circle of subtle subepithelial crystal deposits that appeared to be asymmetric and denser in her left eye (Figure 4). The results for her other tests were normal except for mildly elevated serum total cholesterol and fasting plasma glucose. She had a history of suppurative encephalitis at two months of age.

### Mutation analysis

**Figure 5:**
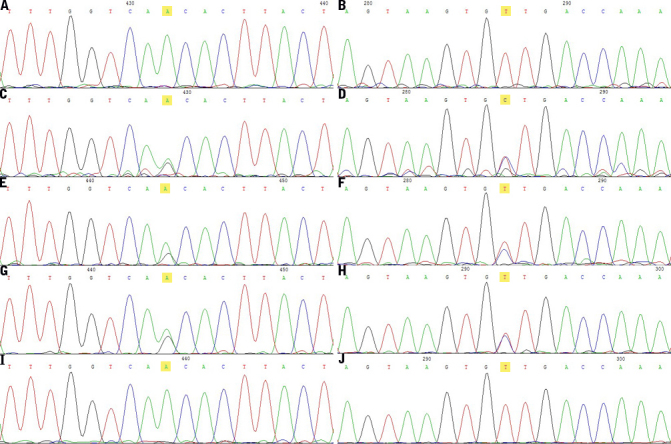
Mutation in *UBIAD1*. Chromatograms of the subjects whose DNA samples were sequenced directly showing heterozygous mutation N102S in exon 1. Normal sequence of *UBIAD1* near codon 102 detected in a healthy control (**A**, **B**) is showed on the first of forward and reverse reading, respectively. The left ones are forward reading, and the right ones are reverse reading. The sequence in the proband (**C**, **D**) shows a heterozygous A>G transversion (at condon 102 leads to a change from asparagine (AAC) to serine (AGC), which is highlighted in yellow), which is also in other affected members: the proband’s daughter (**E**, **F**), and the proband’s granddaughter (**G**, **H**), but not in any unaffected members or normal controls. The indeterminate phenotype member (**I**, **J**) in Figure 1 is determined as unaffected individual due to the chromatograms are the same with the controls (**A**, **B**).

A missense mutation on exon1, c.305A>G (p.Asn102Ser), was identified in the heterozygous state in the proband and affected individuals in whom *UBIAD1* genetic screening was performed (Figure 5). The N102S mutation was shared by the affected members (II:1,III:1,IV:1), and absent in unaffected members and in the 50 unrelated normal controls. The proband’s brother (II:5) of an undetermined affected status has been demonstrated to be an unaffected member because the N102S mutation was not identified.

## Discussion

SCD is a rare corneal dystrophy; there are rare sporadic cases. Although most SCD pedigrees have had European ancestry with Swedish or Finnish origins [17], the dystrophy has also been reported in the Asian population [7,10,11,18]. SCD has been found in Caucasian, Occidental, and African-American populations [1]; in Asia, Chinese, Japanese, Indian, and Saudi Arabian populations have manifested with SCD [1,10,11,14,19]. Reports of SCD in Chinese individuals are rare, and have appeared as single cases or as cases where only a few family members are afflicted.

**Figure 6:**
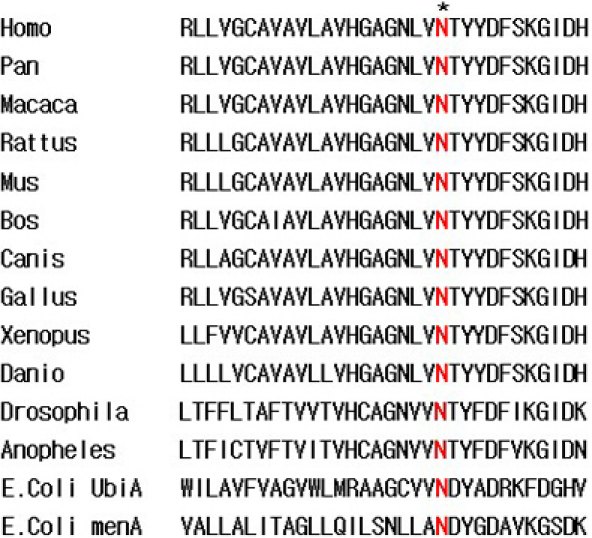
The aligment of UBIAD1 putative orthologs in vertebrate and invertebrate. The N is highly conserved in UBIAD1 proteins from diverse species.

In the present study, we identified a heterozygous missense mutation c.305A>G (p.Asp102Ser), which was confined to the three affected individuals in the SCD pedigree (Figures 1 and Figure 5). The mutation was not found in any unaffected individual in the family, in 50 unrelated controls, or in the Single Nucleotide Polymorphism database (dbSNP), providing evidence to support the hypothesis that SCD is caused by *UBIAD1* mutations. The N102 is a highly conserved gene sequence in putative gene orthologs from other vertebrate and invertebrate genomes (Figure 6). Moreover, it was reported that N102S is the most frequent mutation found in Caucasian SCD patients of either European or unknown ethnicity [13]. Cumulatively, 12/31 (39%) of apparently unrelated families possess this single alteration [9]. These families are of different ethnicities described as British, German, Czechoslovakian, Italian, Irish, Canadian, and American with unknown ethnicity, Chinese (Taiwanese), and Japanese [9,13]. Genetic analysis of families suggested a putative mutational hotspot, i.e., N102S, in Caucasians. To date, in the published literature, all Chinese pedigrees with SCD possessing the N102S mutation are from Taiwan [13]. Our patients in the present study are Han Chinese from a family in the northeast region of mainland China; moreover, they have no ethnic relationship with Caucasians of any background. Hence, the identification of a mutation of the *UBIAD1* gene in this study expands the number of ethnicities for which the spectrum of mutation described earlier in the article is present. As a result of this discovery, a new challenge has presented itself with regard to determining whether the N102S mutation is independent or the result of founder mutation [13]; Nickerson et al. [9] noted that some families with the N102 mutation may be distantly related. The nonsynonymous mutation, N102S, in *UBIAD1* detected in this family confirms that it is a mutation hot spot not only in Caucasians but also in Chinese populations. This finding adds support to the proposal that N102S has independently mutated, and argues against the likelihood of a founder effect.

The *UBIAD1* causative gene for SCD is a highly conserved gene spanning 22 kb localized in chromosome 1p36. The locus contains up to five exons with several different potential transcripts. Bioinformatics analysis suggests that the UBIAD1 protein is an intrinsic membrane protein with a prenyltransferase functional domain and up to eight transmembrane spanning regions [7]. To date, 22 different mutations have been reported in publications, only in exons 1 and 2; in other words, all identified mutations occurred within the predicted prenyltransferase domain. In the predicted two-dimensional model the locations of mutations found in previous research showed several clusters of mutations, which were circled. These mutations were each composed of an aqueous portion and two transmembrane helices, and described as loops 1, 2, and 3. All alterations discovered in amino acids of UBIAD1 faced the other aqueous compartment (i.e., on the other side of membrane). A tertiary model showed transmembrane helices forming a substrate binding cleft on one side of the lipid bilayer. N102S occupied the positions where the first transmembrane helices emerged from the membrane within the putative polyprenyldiphosphate binding site, as identified by Suvarna et al. [20] and Melzer et al. [21], in loop 1 of UBIAD1. More recently, Nickerson et al. [9] identified that both wild-type and the N102S protein were localized subcellularly to mitochondria by immunohistochemistry using antibodies specific for UBIAD1 protein in keratocytes. They also found a putative second substrate of UBIAD1, naphthalin-1, 4-diol, which fitted well into the binding pocket and docked preferentially in the central cavity in close proximity to amino acid, N102. The 3-D model showed the N102 residue, a spacefill atom with a docked farnesyldiphosphate, the sidechain of which pointed inwards toward the center of a speculated prenyldiphosphate binding pocket. The mutation, N102S, had completely changed binding of the substrate, and prenylation at position 3 was no longer possible. Thus, it is speculated that mutated N102 residue plays a critical role in SCD, which would block the critical steps in catalysis suggested by UBIAD1 substrate docking models [9]. The locations of mutation clusters are interesting; whether all of the mutations have a similar effect and whether the function of the mutated protein is up- or down-regulated are still not resolved, though it has been indicated by Nickerson et al. [9] that the activity of mutated protein appeared to be down-regulated. Moreover, additional experiments are necessary to explain the amino acid alternations on one side of the lipid bilayer, and to identify the actual ligand that binds UBIAD1. Further researches need to be undertaken to determine whether these mutations affect protein folding and/or targeted for degradation, and whether other mutations like N102S were localized subcellularly in the same fashion as the wild-type proteins.

An important distinguishing feature of SCD is that it is associated with a systemic manifestation, hypercholesterolemia, which is found in approximately 66% of affected patients [3-5]. Our findings in this pedigree (2 of 3 patients had hypercholesterolemia) are consistent with this discovery. Both affected and unaffected individuals in the family with SCD may have hyperlipoproteinemia (type IIa, III, or IV) [2]. Serum lipid, lipoprotein, or cholesterol levels may be normal or abnormal in the patients; this is also true for unaffected members of the pedigree [3,18,22-24]. Affected members of a pedigree demonstrate a higher prevalence of hypercholesterolemia than does the general population [23]. In this Chinese SCD pedigree, the majority of family members who were examined included patients with abnormal lipid metabolism (Table 1). Even though many patients with SCD have dyslipidemia, there is no relationship between the severity of the dyslipidemia and the occurrence of crystal formation, according to overall consensus [25]. Meanwhile, it is considered that the progress of corneal opaciﬁcation is not associated with serum lipid levels [26,27]. In spite of the knowledge already gathered, the pathogenesis of SCD is unclear. SCD is presumed to be due to a localized lipid metabolism defect in the cornea. It is postulated that one possible reason for SCD is the overproduction of cholesterol according to the *UBIAD1* gene producing a protein with a prenyltransferase domain, which plays a role in cholesterol metabolism. Another possible cause is a defect in the removal of cholesterol in view of the interaction of UBIAD1 and the COOH-terminal portion of apolipoprotein E, which mediates removal of cholesterol from cells [13]. Recently, Nickerson and colleagues  [9] analyzed cholesterol metabolites in patient cell line extracts. This analysis showed no significant alteration in the presence of mutant protein, indicating a potentially novel function of the UBIAD1 protein in cholesterol biochemistry [9]. More experimental studies will determine whether the excess cholesterol in the cornea results from increased cholesterol production or decreased cholesterol removal.

**Table 1:** Biochemical findings.

**Individual**	**Age**	**TC**	**TG**	**HDL-C**	**LDL-C**	**Calcium**	**Glucose**	**Apo B**
II:1	57	↑	N	N	N	↓	N	/
II:2	58	N	↑	N	N	N	N	/
II:4	56	N	N	↑	N	↓	N	↓
II:5	54	↑	N	↑	N	N	↑	N
II:8	51	↑	N	↑	↑	↓	↑	N
III:1	34	N	N	N	N	N	↓	/
III:3	24	↑	↑	N	↑	↓	N	/
III:4	30	N	N	↑	N	↓	N	N
III:7	26	N	N	↑	N	↓	N	↓
III:10	26	N	N	↑	N	↓	N	↓
IV:1	11	↑	N	N	N	N	↑	/

Arrow upward indicates which comparing to normal is higher, conversely arrow downward is used, and N is shot for normal. The sprit indicates that there is no result obtained. TC=total cholesterol; TG=triglyceride; HDL-C=high density lipoprotein cholesterol; LDL-C=low density lipoprotein cholesterol; Apo B=apolipoprotein B.

The clinical findings in the patients identified in this study are consistent with corneal appearance predicted based on age, as previously described [28]. Patients younger than 23 years demonstrate only a central corneal opacity with or without central subepithelial cholesterol crystals and possess excellent visual acuity and normal corneal sensation (Patient IV:1, Figure 4). Patients aged 23–39 years develop arcus lipoides, and visual acuity may be diminished (Patient III: 1, Figure 3). In patients older than 39 years, a mid-peripheral, panstromal corneal haze appears that fills in the areas between the central opacity and the peripheral arcus (which could be seen without a slit lamp), and an objective loss of visual acuity and reduced corneal sensation (the proband, Figure 2). The decreased corneal sensation in the proband could be explained by what has been found in confocal microscopy examination: intracellular and extracellular highly reflective deposits leading to eventual disruption of the basal epithelial/subepithelial nerve plexus [2].

There was no genotype–phenotype correlation for the majority of mutations; phenotypic variation was present within families. We found affected individuals shared the same N102S mutation, but had very different corneal appearance (Figure 2, Figure 3, and Figure 4). Affected individuals from different families possessed different mutations, but had virtually identical corneal ﬁndings, as described in the literature. It is presumed that modulating inﬂuences such as environmental effects lead to the observed phenotypic heterogeneity, and that the interaction of multiple genes resulted in a speciﬁc phenotype [13].

Interestingly, we identified 7 of 10 family members (70%) in whom blood biochemical examinations (Table 1) showed decreased serum calcium levels, while levels of serum phosphorus were normal. Serum calcium and phosphorus levels were also normal in the spouse of the proband. As yet, no relationship between SCD or *UBIAD1* and calcium metabolism has been described in the literature; hence there is no inconsistency between our results and other published work. By consulting a broad range of articles, we speculate that *UBIAD1* may impact the serum calcium level through an effect on the vitamin K-dependent enzyme and its derivatives. A recent study found that UBIAD1 was a human Menaquinone-4 (MK-4, i.e., vitamin K_2_) biosynthetic enzyme [29]. In fact the activity of vitamin K encompasses many kinds of physiologic processes, including blood coagulation, regulation of tissue calcium content [30], and gene activity [31]. Vitamin K also has effects on growth regulation [32], anti-inflammation [33], anti-canceration [34], and antioxidation [35]. Coincidentally, the location for vitamin K recycling—a very crucial process for its biologic action [30]—and the location of UBIAD1 protein (of osteoblast cells) [29] are both in the endoplasmic reticulum membrane. Moreover, it was demonstrated that vitamin K is a cofactor in bone metabolism [36,37]. Vitamin K has activity in the post-translational modification of vitamin K-dependent proteins, which involves the conversion of glutamic acid residues to gamma-carboxyglutamic acid residues, enables the enzyme to carboxylate selected proteins in targeted glutamate groups. In the process, vitamin K functions as a cofactor with gamma-glutamyl carboxylase. In addition, vitamin K_2_ has the most potent gamma-carboxylation activity [38]. Some vitamin K-dependent proteins, such as calbindin and osteocalcin, are also calcium-binding proteins that play a role in calcium homeostasis and in facilitating bone mineralization [39]. Furthermore, some recent studies reveal that osteocalcin is involved in metabolism in both bone metabolism and energy metabolism, including glucose and lipid metabolism.

Osteocalcin is expressed in many tissues, secreted from osteoblasts, and produced and regulated by 1,25-dihydroxyvitamin D, glucocorticoids, estrogens, and retinoic acid [39]. Osteocalcin exists in two forms: carboxylated (this form accounts for most osteocalcin) and undercarboxylated. Carboxylated osteocalcin is essential for bone mineralization, which binds calcium strongly and consolidates calcification of the hydroxyapatite crystal lattice in bone; some osteocalcin is undercarboxylated and is located in the circulation, where it involves in the regulation process such as glucose metabolism, lipid metabolism, and insulin secretion and sensitivity. Other research found that serum osteocalcin was associated with metabolism of lipids, including triglycerides, cholesterol, and high-density-lipid cholesterol (HDL-C) [40]. Table 1 shows the number of members of the current pedigree with high HDL-C levels. Serum calcium is lower in patients after obesity surgeries, with the serum osteocalcin level changing while the phosphorus level remains normal [41]. Interestingly, both the osteocalcin gene and *UBIAD1* are located on chromosome 1. There may be an as-yet incompletely characterized, complicated relationship between osteocalcin and *UBIAD1* in SCD.

In summary, it is possible that some unknown has not been identified in SCD, and in the function of *UBIAD1* to explain serum calcium in low levels. More studies are needed to determine whether serum calcium or osteocalcin is associated with SCD or with *UBIAD1*.
